# Clinical validation of the Swaasa® artificial intelligence platform using cough sounds for screening and aid in diagnosis of respiratory diseases

**DOI:** 10.3389/fmed.2026.1964851

**Published:** 2026-10-28

**Authors:** Balamugesh Thangakunam, Sujith Thomas Chandy, Shubha Deepti Palreddy, Prasanna Samuel P., Devasahayam Jesudas Christopher

**Affiliations:** 1 Department of Pulmonary Medicine, Christian Medical College, Vellore, India; 2 Salcit Technologies, Kothaguda, India; 3 Department of Biostatistics, The Tamil Nadu Dr. M.G.R. Medical University, Chennai, India

**Keywords:** airflow parameters, cough sound characteristics, machine learning, respiratory conditions, respiratory diseases

## Abstract

**Introduction:**

Cough sound analysis can provide clues to underlying respiratory disease. The Swaasa AI platform, a Software as a Medical Device (SaMD), applies artificial intelligence to cough recordings to generate indications, including overall risk (normal vs. abnormal), spirometric pattern (obstructive, restrictive, or normal), pattern severity (mild, moderate, severe), and the presence of asthma, COPD, ILD, and bronchiectasis.

**Methods:**

Patients (≥18 years) presenting to our departments with respiratory symptoms were prospectively recruited. Cough sounds were recorded on a mobile device running the Swaasa AI platform, spirometry was performed. Spirometry served as the reference standard for physiological pattern and severity, while physician-established clinical diagnosis served as the reference for disease categories. Polychotomous variables were dichotomized as normal vs. abnormal, & multi-class output for disease condition, pattern severity, and disease categories (asthma, COPD, ILD, bronchiectasis) were defined.

**Trial registration:**

CTRI/2021/04/032742; registered on 12/04/2021; date of first enrollment: 19/04/2021; trial end date: 14/09/2023.

**Results:**

We recruited 2,179 patients with respiratory diseases and 827 normal individuals; 62% were male. The Swaasa AI platform achieved 90% accuracy in distinguishing normal from abnormal (respiratory condition present) and in classifying normal vs. abnormal spirometry, with descriptive performance subgroup analyses showing broadly comparable performance across age and gender groups For multi-class outcomes, the accuracy ranges were: spirometry pattern: 79%–84%, pattern severity: 76%–84.5%, and disease-type classification: 83%–93%.

**Conclusion:**

Swaasa® demonstrates promising performance in distinguishing normal from abnormal respiratory conditions across demographic subgroups, indicating its promise as a screening tool. While the accuracy measures emphasize further refinement to ensure consistent high performance across specific respiratory disease scenarios and severity levels, the study shows its reasonable potential in identifying specific respiratory diseases and their severity.

## Introduction

Respiratory sounds generated during coughing and breathing contain clinically relevant information for respiratory diseases, and modern technology enables their simple, non-invasive acoustic recording, thereby improving their utility in screening and clinical assessment (1).

Recent digital technological advances have enabled sophisticated systems for recording and analyzing cough sounds. These have been evaluated for screening, triage, and treatment monitoring in asthma, Chronic Obstructive Pulmonary Disease (COPD), and post-COVID–19 states (2–6). Cough sound devices quantify cough intensity and frequency and delineate disease-specific cough signatures (7–9).

Respiratory diseases comprise conditions that weaken normal lung function. In clinical practice, pulmonary function testing (PFT) provides key information on lung status and supports the diagnosis of respiratory disease (10, 11). Spirometry is one of the most accessible and informative PFT modalities (12).

Spirometry measures the volume of air exhaled at specific time points during a forced expiration following maximal inhalation (13). Patients inhale deeply and exhale forcefully through a mouthpiece, with the maneuver repeated until three consistent efforts are obtained under the supervision of trained respiratory therapists (14). Reference values for forced expiratory volume in 1 s (FEV1), forced vital capacity (FVC), and the FEV1/FVC ratio are derived from age, sex, and height. The ratios of measured to reference values (FEV1% and FVC%) indicate disease severity (15). FEV1/FVC and FVC% differentiate obstructive, restrictive, and normal patterns, whereas FEV1% grades the severity of obstructive disease (16).

Recently, home-based spirometry for monitoring lung function in asthma, Chronic Obstructive Pulmonary Disease (COPD), and interstitial lung disease (ILD) has gained prominence in clinical practice and research (17–21). It improves convenience, accessibility, and frequency of lung function assessment, further supported by smartphone applications that enhance patient–provider communication. Advances in machine learning (ML) and expanding health datasets are enabling improved quality and interpretation of pulmonary function testing (22, 23), although most ML research to date has focused on automating existing diagnostic tasks.

Traditional diagnostic tools for respiratory diseases—such as spirometry, swab tests, bronchoscopy, and radiological imaging—are effective but often limited by accessibility and cost, highlighting the need for alternative approaches. Artificial intelligence (AI), particularly machine learning, offers more accessible, cost-efficient screening and clinical assessment using widely available devices like smartphones.

The Swaasa AI platform uses a 10 s cough recording collected through a smartphone/tablet, with the device positioned 4–8 inches from the mouth at a 90° angle. Noise reduction was applied using multi-microphone signal processing or FFT-based noise subtraction for single-microphone devices. A cough/non-cough classifier identified valid cough events and excluded silence, speech and background noise. Cough events were typically 200–700 ms, with approximately 20 ms analysis frames. The system extracted time- and frequency-domain features, including ZCR, energy, MFCCs, spectral centroid, bandwidth and roll-off. Initially, 209 features were extracted and reduced to 170 primary features using correlation-based feature selection/RFE; secondary features included age, gender, symptoms, cough type and cough duration. The model uses two parallel architectures: a ResNet-34 CNN with ImageNet transfer learning operating on Mel spectrograms, and an FFANN operating on the primary and secondary tabular features. The FFANN contains two hidden layers with 400 and 300 neurons, followed by batch normalization, and the outputs of both models are merged through a final fully connected combined logic layer.

Our research investigates cough sound signal analysis, aiming to map these signals to their corresponding respiratory diseases and evaluate cough as a respiratory screening and clinical assessment, focusing on disease-specific acoustic features and inter-disease differentiation. On the other hand, cough recording requires minimal training, no physical contact, and is effective for disease assessment (17–24), and clinical evidence indicates that cough severity correlates with respiratory disease progression (25).

Signal processing enables the extraction of clinically relevant features from cough sounds, and prior work has highlighted the value of cough classification for detecting respiratory illnesses (25). Our previous studies demonstrated strong correlations between cough sound characteristics and airflow parameters (FEV1, FVC, and their ratios), which are central to lung disease classification (7). Building on this, the present study evaluates the Swaasa AI platform (https://swaasa.ai/) for high-precision classification of normal and abnormal conditions across demographic subgroups, supporting its potential as a screening tool.

## Methods

### Data collection

All patients were examined by a respiratory physician and, on the same day, underwent spirometry and cough sound assessment via a mobile phone shown in Figure 1.

**Figure 1:**
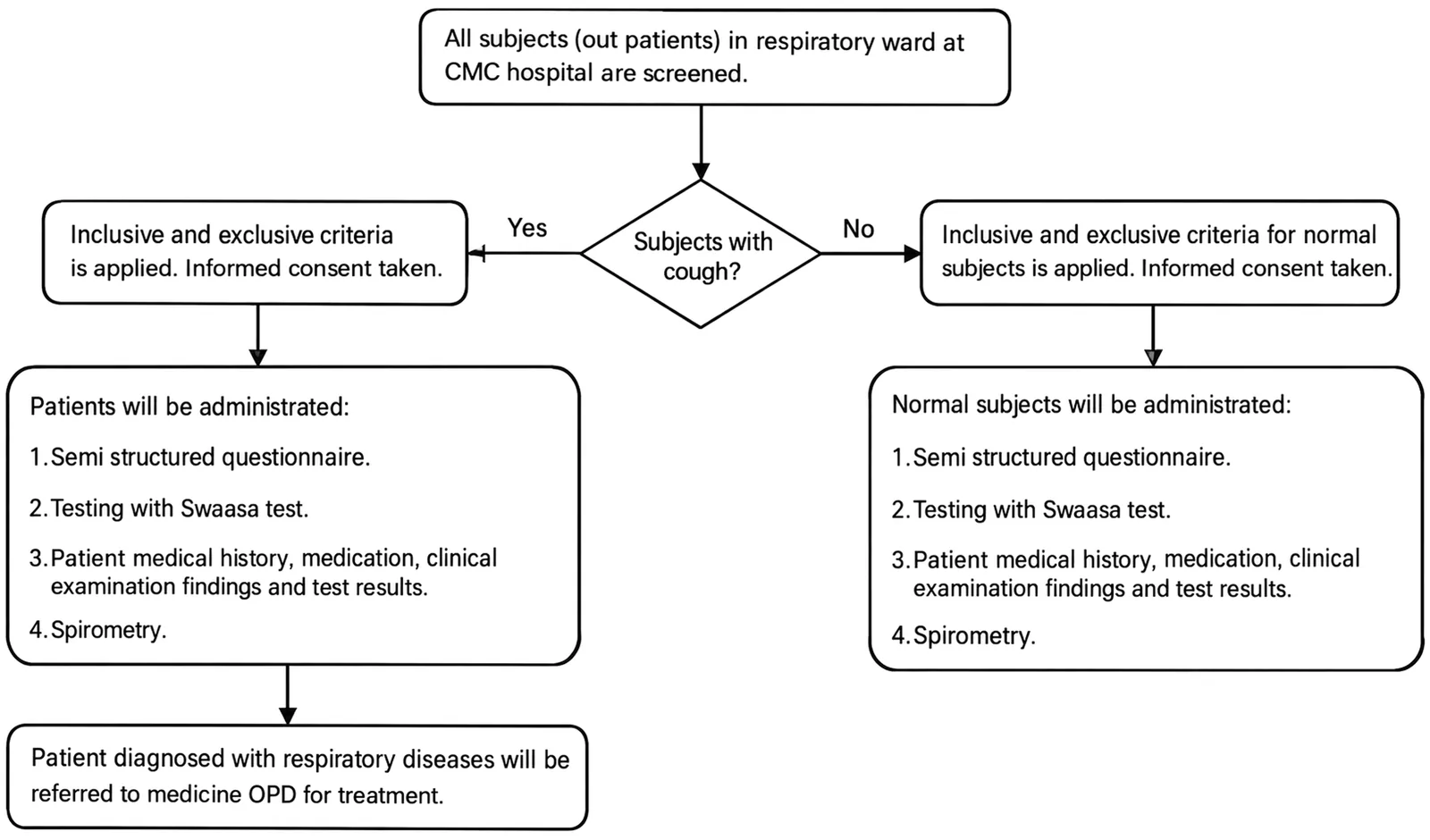
Data collection process.

Demographic data, vital signs, and a subset of the SGRQ questionnaire were collected to characterize respiratory symptoms and lung health (26). Informed consent was obtained from all participants and/or their legal guardians. Cough sounds were recorded using the in-built mobile microphone of the Swaasa AI platform, with five voluntary coughs per participant; due to noise, invalid, or void recordings, an average of three coughs per participant were retained as individual datapoints. The first retained recording from each participant was used for participant-level analysis, ensuring one independent observation per participant. Subsequent recordings were considered repeated recording-level observations and were used to assess algorithm performance and device reliability across repeated cough samples. A minimum interval of 10 s and a maximum of 3 min between coughs was maintained to allow adequate receptor triggering, and multiple recordings per participant were used to assess device reliability.

Spirometry was performed according to the 2019 American Thoracic Society guidelines (27). Abnormal results were categorized as obstructive or restrictive based on the FEV1/FVC ratio, with isolated MMEF reductions classified separately. For accuracy analyses, for physiological pattern classification (normal, obstructive, restrictive, and mixed) and severity assessment, the reference was based on spirometry findings and their clinical interpretation. For disease-specific outputs (asthma, COPD, ILD, and bronchiectasis), the reference was the final physician-assigned clinical diagnosis. For Asthma, COPD, bronchiectasis diagnosis is based on existing international guidelines GINA, GOLD, ERS. ILD diagnosis based on multidisciplinary team discussion.

Spirometry was selected as the reference standard for physiological pattern and severity classification because it provides objective measurements of airflow limitation and lung function that are directly relevant to categorizing obstructive/restrictive patterns and their severity. In contrast, disease categories such as asthma, COPD, bronchiectasis, and ILD cannot be established by spirometry alone and therefore relied on the treating physician's clinical diagnosis, based on the available clinical history, examination, spirometry, imaging, and other relevant investigations. We acknowledge the limitations of these reference standards. Spirometry is effort- and technique-dependent and may not fully characterize all respiratory abnormalities, while clinician-assigned diagnoses may involve clinical judgement and variability in the investigations available for individual patients.

Participants meeting the predefined eligibility criteria were consecutively recruited during the study period. The procedure was double-blinded: neither the participants, therapists, nor physicians had access to the Swaasa® report.

### Sample size

For sample size calculation, assuming the device would achieve 80% sensitivity and specificity with a 5% error margin at 95% CI for detecting respiratory conditions, and a disease prevalence of 25%, a sample of 990 participants was deemed adequate for validation. To enhance rigor and target 90% sensitivity/specificity, the sample size was increased to 1,300, with one-fourth allocated to an additional derivation cohort. Allowing for incomplete data and inability to perform spirometry, we aimed to recruit 1,800 participants, with 38% normal controls and the remainder evenly distributed across asthma, COPD, ILD, and bronchiectasis. Normal subjects were asymptomatic relatives accompanying patients to the outpatient clinic. Ultimately, 1,917 participants were available for the final validation analysis.

The sample-size calculation was performed at the participant level based on the prespecified assumptions for sensitivity, specificity and disease prevalence. The calculated sample-size requirement therefore refers to unique participants. Repeated recordings were treated as repeated algorithm assessments and were not considered additional independently recruited participants for the purpose of sample-size determination.

Patients and members of the public were not involved in the design, conduct, reporting, or dissemination plans of this research.

### Ethical considerations

The study was registered under Clinical Trials Registry- India (CTRI/2021/04/032742) and was begun after getting the approval [Ethics Approval number - IRB Min. No. 13566 (DIAGNO)] from the Office of Research, Institutional Review Board (IRB), Christian Medical College, Vellore, India. And all research was performed in accordance with relevant guidelines/regulations.

The study protocol is not currently available in a public repository. The protocol may be made available upon reasonable request to the corresponding author, subject to applicable institutional and ethical requirements.

This study was conducted in accordance with the principles of the Declaration of Helsinki.

### Study design

This monocentric, exploratory study aimed to validate the Swaasa AI platform in a real-world cohort of patients with respiratory conditions at the Respiratory and Pulmonary Medicine departments of Christian Medical College (CMC), Vellore.

The statistical validation focused on estimating the accuracy of Swaasa® in:

(1) Classifying risk as normal vs. abnormal
(2) Identifying respiratory disease pattern and severity, and
(3) Detecting disease conditions in asthma, COPD, ILD, and bronchiectasis.

The validation used data from a prospective diagnostic accuracy study and followed two plans. Plan 1 assessed risk (normal vs. abnormal), pattern, and severity, while Plan 2 focused on disease classification. These plans represent different strategies for organizing data for AI model training and testing. In both, subject data did not overlap between the derivation and validation cohorts.

The validation model, including its inputs and outputs, is shown in Figure 2.

**Figure 2:**
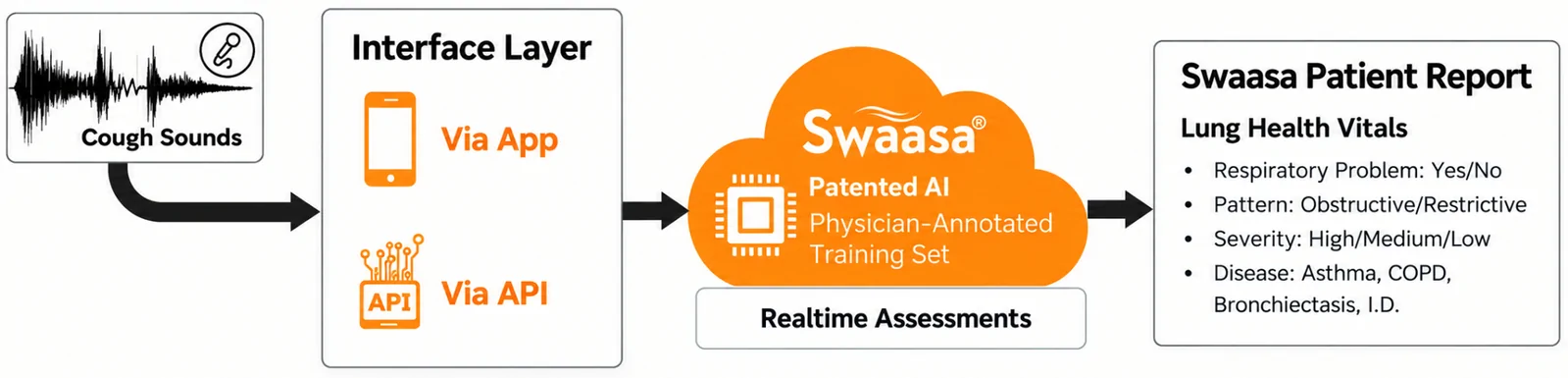
A flowchart highlighting the steps of swaasa AI platform.

Pre-processing methods and model architectures have been described previously (7–9). Briefly, Swaasa® analyzes digitally recorded cough sounds using acoustic features extracted from both the time and frequency domains. These include zero-crossing rate (ZCR), energy, Mel-frequency cepstral coefficients (MFCCs), spectral centroid, spectral bandwidth, and spectral roll-off, along with other acoustic descriptors used in the condition-specific models (8, 9). The cough-sound information is processed using a convolutional neural network (CNN), while relevant tabular/clinical features are processed using a feedforward artificial neural network (FFANN); the outputs of these complementary models are combined to generate the screening result (8, 9). The underlying models were developed and validated on clinically collected datasets, with separate validation cohorts used to assess model performance. Clinicians labeled respiratory conditions, providing the gold-standard annotations for evaluating the AI platform. Performance for binary and multi-class outcomes was assessed using accuracy, kappa statistics, and diagnostic accuracy measures (sensitivity and specificity), under two analysis plans (Plan 1 and Plan 2) tailored to validate specific models.

The study followed a sequential derivation-validation approach. During the model-development phase, data from 450 participants in Plan 1 and 515 participants in Plan 2 were incorporated into the existing development dataset, which also included previously available clinical datasets. These participants formed part of the derivation/development cohort and were used, as applicable, for model development and finalization of the decision thresholds.

Following completion of this derivation phase, the model parameters and decision thresholds were finalized and locked before evaluation of the validation cohort. The participants included in the reported validation analyses were separate from the derivation participants and were not used for model training, feature selection, calibration, threshold selection, or subsequent model tuning.

Plan 1—Risk, Spirometry Pattern, Pattern Severity

○ Design: Validation of models for risk (normal vs. abnormal), spirometry pattern, and pattern severity
○ Total subjects: 1,917
○ Derivation cohort: 450 subjects (added to existing training dataset)
○ Validation cohort: 1,467 subjects

Plan 2—Disease Models

○ Design: Validation of disease classification models
○ Total subjects: 1,917
○ Derivation cohort: 515 subjects (integrated into training dataset)
○ Validation cohort: 1,402 subjects

Figure 3 shows the data distribution by age and sex.

**Figure 3:**
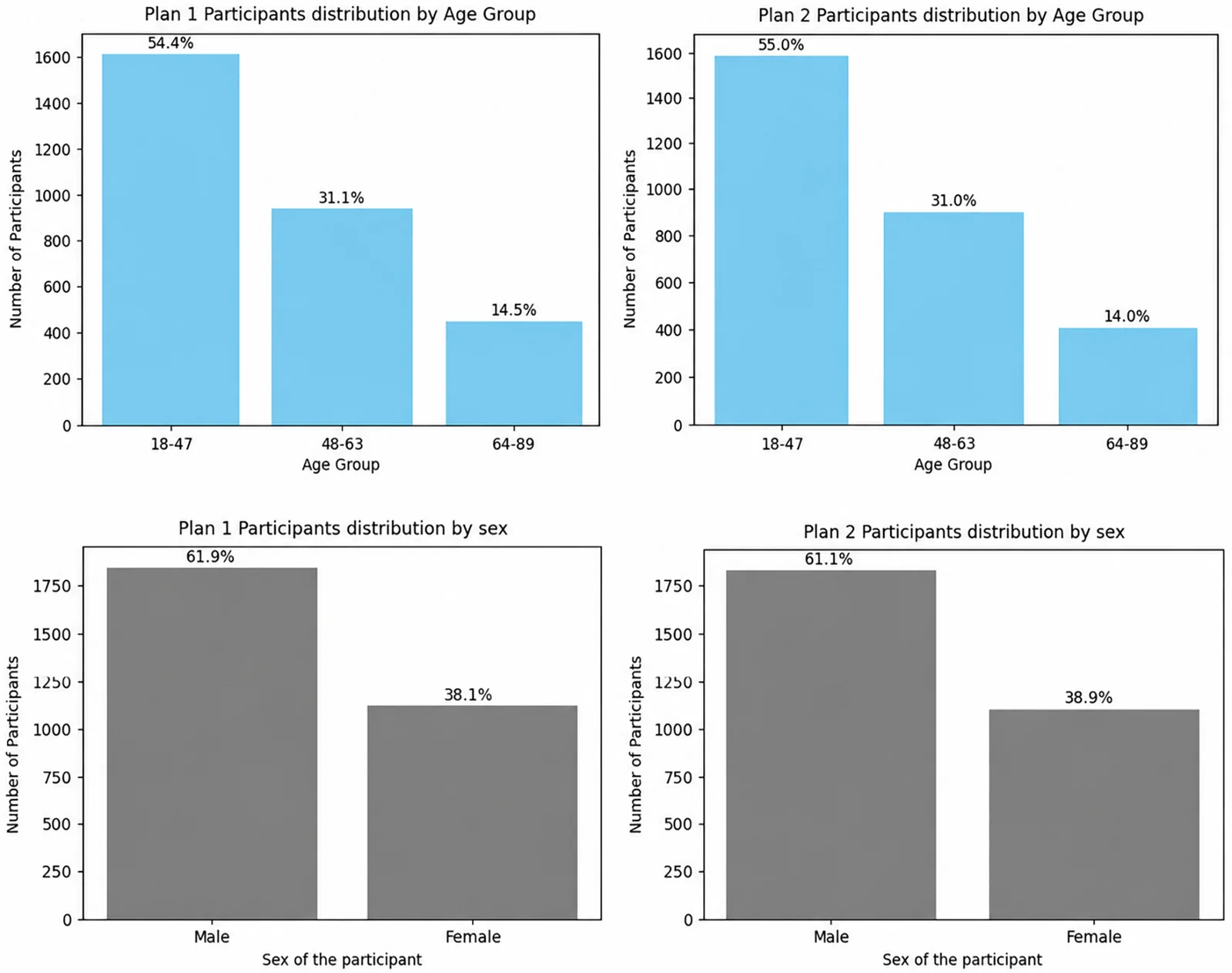
Data distribution in plan 1 and plan 2.

This study was reported in accordance with the STARD 2015 guideline for diagnostic accuracy studies. The completed STARD 2015 checklist is provided as Supplementary Material.

## Results

### Participants' information

- Three cough audio recordings were collected from each participant.
- Plan 1: 3,006 cough recordings from 1,467 subjects.
- Plan 2: 2,832 cough recordings from 1,402 subjects.
- Table 1 summarizes the distribution of clinical and demographic variables.
- Demographic, clinical, and other variables of interest were summarized using frequency tables.

**Table 1:** Distribution of clinical and demographic variables.

Variables	Data available for validation	
	*n* (%)	*n* (%)
	*N* = 3,006 (Plan 1)	*N* = 2,832 (Plan 2)
Age group		
18–47 years	1,636 (54.4)	1,559 (55.0)
48–63 years	934 (31.1)	877 (31.0)
64–89 years	436 (14.5)	396 (14.0)
Sex		
Male	1,862 (61.9)	1,730 (61.1)
Female	1,144 (38.1)	1,102 (38.9)

The 3,006 observations in Plan 1 and 2,832 observations in Plan 2 represent individual cough recordings obtained from these participants and not additional participants. Multiple recordings were collected from the same participant at a minimum interval of 10 s and a maximum of 3 min between recordings, with each recording independently processed by the Swaasa® algorithm. As the underlying clinical condition and reference-standard diagnosis were unchanged during this period, the same participant-level clinical reference label was applicable to the respective recordings.

### Comparison of Swaasa® results with ground truths

To evaluate the diagnostic accuracy of Swaasa® as a screening tool, confusion-matrix-based statistics were used, including accuracy, kappa, sensitivity, and specificity. Accuracy, the proportion of correct predictions among all predictions, provides an overall measure of model performance. Tables 2, 3 compare Swaasa® outputs with the ground truth. Kappa quantifies agreement between the model and observed data, sensitivity (true positive rate) reflects the ability to correctly identify positive cases, and specificity (true negative rate) reflects the ability to correctly identify controls. Together, these metrics provide a comprehensive assessment of the model's classification performance.

**Table 2:** Frequencies for the ground truth and Swaasa® risk, spirometry pattern variables with binary classification.

Outcomes	Ground truth (n%)	Swaasa® (n%)
Risk		
Normal	827 (27.5)	835 (27.8)
Abnormal	2,179 (72.5)	2,171 (72.2)
Total	3,006 (100.0)	3,006 (100.0)
Spirometry pattern (Normal vs. Abnormal)		
Normal	1,486 (49.4)	1,377 (45.8)
Abnormal	1,214 (40.4)	1,639 (54.2)
Missing*	306 (10.2)	NA
Total	3,006 (100.0)	3,006(100.0)

*This data was not used for validation.

**Table 3:** Frequencies for ground truth and Swaasa® and disease variables with multiple classes swaasa AI platform predicts risk, spirometry pattern, pattern severity and disease type from cough audio recording.

Outcomes	Ground truth (n%)	Swaasa® (n%)
Diagnosis disease type		
Normal	852 (30.1)	857 (30.3)
Asthma	510 (18.0)	746 (26.3)
Bronchiectasis	80 (2.8)	286 (10.1)
COPD	252 (9.0)	577 (20.4)
ILD	156 (5.5)	365 (12.9)
Multi—labelled	845 (29.8)	NA
Missing*	137 (4.8)	1 (0.0)
Total	2,832 (100.0)	2,832(100.0)

*This data was not used for validation.

Risk: Risk denotes the presence of an underlying respiratory abnormality. Swaasa® classifies risk as “Abnormal” when any abnormality is detected and “Normal” otherwise.

Spirometry Pattern: Physiological pattern of lung function based on spirometry (e.g., obstructive, restrictive, or normal), typically derived from FEV1, FVC, and FEV1/FVC.

Pattern Severity: Degree of impairment within a given spirometric pattern (e.g., mild, moderate, severe), commonly graded using FEV1% and/or FVC% relative to reference values. The severity categories include “Normal”, “Mild”, “Moderate,” and “Severe.” The spirometry report values are used as a reference to generate ground-truth data and to build the severity model. When the spirometry report says normal, the ground truth of the patient is considered to be Normal. The severity categories are mapped to the FEV1% predicted values such as: Mild (>70), Moderate (70–50), Severe (<50) (28).

Disease Model: It focuses on detecting various respiratory conditions. Swaasa® outputs diseases as “Asthma,” “COPD,” “ILD,” “Bronchiectasis,” and “Normal.” In the case of Abnormal vs. Normal detection, all diseases, including multi-labelled values from the ground truth, are considered abnormal.

### Performance metrics of the Swaasa® AI models

The results demonstrate Swaasa's ability to distinguish normal from abnormal cases across the major assessment domains, supporting its potential as a screening tool. Crosstabulations for risk, pattern, and severity are presented in Tables 4–7. These findings further indicate reasonable performance in identifying specific respiratory diseases and their severity.

**Table 4:** Cross tabulation of Swaasa® risk and underlying respiratory problem with binary classification along with column percentages.

Swaasa® risk	Underlying respiratory problem		
	Normal	Abnormal	Total
Normal	667 (80.7)	168 (7.7)	835 (27.8)
Abnormal	160 (19.3)	2,011 (92.3)	2,171 (72.2)
Total	827 (100)	2,179 (100)	3,006 (100)

**Table 5:** Cross tabulation of Swaasa® and ground truth spirometry pattern with multiple classes along with column percentages.

Swaasa® Pattern	GT Spirometry Pattern					
	Normal	Obstructive	Restrictive	MMEF	Missing	Total
Normal	639 (85.6)	83 (12.2)	96 (18.0)	458 (61.9)	101 (33.0)	1,377 (45.8)
Obstructive	96 (12.9)	553 (81.2)	99 (18.6)	219 (29.6)	131 (42.8)	1,098 (36.5)
Restrictive	11 (1.5)	45 (6.6)	338 (63.4)	63 (8.5)	74 (24.2)	531 (17.7)
Total	746 (100)	681 (100)	533 (100)	740 (100)	306(100)	3,006(100)

**Table 6:** Crosstabulation of Swaasa® and ground truth pattern severity with multiple classes along with column percentages.

Swaasa® Pattern Severity	GT Pattern Severity					
	Normal	Mild	Moderate	Severe	Missing*	Total
Normal	479 (60.9)	256 (24.2)	67 (13.9)	18 (4.8)	26 (8.5)	846 (28.1)
Mild	68 (8.7)	586 (55.4)	16 (3.3)	14 (3.7)	20 (6.5)	704 (23.4)
Moderate	109 (13.9)	79 (7.5)	283 (58.7)	31 (8.3)	128 (41.8)	630 (21.0)
Severe	130 (16.5)	137 (12.9)	116 (24.1)	311 (83.2)	132 (43.1)	826 (27.5)
Total	786 (100)	1,058 (100)	482 (100)	374 (100)	306 (99.9)	3,006 (100)

*This data was not used for validation.

**Table 7:** Cross tabulation of Swaasa® and ground truth disease type pattern with multiple classes along with column percentages.

Swaasa® Disease	Diagnosis Disease Type							
	Normal	Asthma	Bronchiectasis	COPD	ILD	Multi labelled	Missing*	Total
Normal	703 (82.5)	25 (4.9)	8 (10.0)	12 (4.8)	8 (5.1)	69 (8.2)	32 (23.4)	857 (30.3)
Asthma	98 (11.5)	348 (68.2)	10 (12.5)	28 (11.1)	16 (10.3)	226 (26.7)	20 (14.6)	746 (26.3)
Bronchiectasis	13 (1.5)	18 (3.5)	42 (52.5)	35 (13.9)	24 (15.4)	139 (16.4)	15 (10.9)	286 (10.1)
COPD	16 (1.9)	100 (19.6)	12 (15.0)	154 (61.1)	28 (17.9)	226 (26.7)	41 (29.9)	577 (20.4)
ILD	21 (2.5)	19 (3.7)	8 (10.0)	23 (9.1)	80 (51.3)	185 (21.9)	29 (21.2)	365 (12.9)
Missing*	1 (0.1)	0 (0.0)	0 (0.0)	0 (0.0)	0 (0.0)	0 (0.0)	0 (0.0)	1 (0.0)
Total	852 (100)	510 (99.9)	80 (100)	252 (100)	156 (100)	845 (99.9)	137 (100)	2,832 (100)

*This data was not used for validation.

Tables 4–15 present performance analyses based on pooled cough recordings, including repeated recordings from individual participants. Tables 16, 17 additionally present performance separately for Records 1, 2, and 3. The Record 1 analysis in Tables 16, 17 includes only the first retained recording from each participant and therefore represents participant-level performance, with each participant contributing a single independent observation. Records 2 and 3 represent additional repeated recording-level assessments and are presented to evaluate algorithm performance across repeated cough samples.

**Table 8:** Confusion matrix statistics for spirometry pattern, spirometry severity and disease with multi-class variables with 95% confidence intervals.

Parameters	Accuracy (%)	Kappa Statistic (%)	Sensitivity (%)	Specificity (%)	PPV (%)	NPV (%)	F1
Spirometry Pattern							
Normal	79.0 (77.4–80.5)	58.2 (55.2–61.3)	73.8 (71.5–76.0)	85.3 (83.2–87.2)	86.0 (84.0 –87.8)	72.7 (70.3 –75.0)	0.79 (0.78 –0.81)
Obstructive	79.9 (78.4–81.4)	53.3 (49.9–56.6)	81.2 (78.1–84.0)	79.5 (77.7–81.2)	57.2 (54.0 –60.3)	92.6 (91.3–93.8)	0.67 (0.64 –0.69)
Restrictive	88.4 (87.1–89.5)	61.2 (57.3–65.1)	63.4 (59.2–67.4)	94.5 (93.5–95.4)	74.0 (69.7 –77.8)	91.3 (90.1–92.4)	0.68 (0.65 –0.72)
Pattern Severity							
Normal	76.0 (74.4–77.6)	42.6 (38.9–46.3)	60.9 (57.5–64.3)	82.2 (80.4–83.8)	58.4 (55.0 –61.7)	83.7 (81.9 –85.3)	0.59 (0.57 –0.63)
Mild	78.9 (77.3–80.4)	52.7 (49.5–56.0)	55.4 (52.4–58.4)	94.0 (92.8–95.1)	85.7 (82.8 –88.1)	76.6 (74.7 –78.4)	0.67 (0.65 –0.69)
Moderate	84.5 (83.1–85.8)	48.1 (43.8–52.3)	58.7 (54.3–63.0)	90.1 (88.8–91.3)	56.4 (52.0 –60.6)	90.9 (89.7 –92.1)	0.57 (0.54 –0.61)
Severe	83.5 (82.0–84.8)	49.1 (45.2–53.0)	83.2 (79.0–86.6)	83.5 (82.0–85.0)	44.8 (41.2 –48.5)	96.9 (96.0 –97.5)	0.58 (0.55 –0.62)
Diagnosis Disease Type							
Normal	89.1 (87.6–90.5)	77.9 (75.1–80.8)	82.6 (79.9–85.0)	94.7 (93.1–95.9)	93.0 (90.9 –94.6)	86.5 (84.3 –88.4)	0.875 (0.86 –0.89
Asthma	83.0 (81.2–84.7)	57.2 (53.0–61.4)	68.2 (64.1–72.)	88.6 (86.8–90.2)	69.6 (65.4 –73.5)	88.0 (86.1 –89.6)	0.69 (0.65 –0.72)
Bronchiectasis	93.1 (91.8–94.1)	36.2 (27.6–44.8)	52.5 (41.7–63.1)	94.9 (93.8–95.8)	31.8 (24.5 –40.2)	97.8 (97.0 –98.4)	0.4 (031 –0.48)
COPD	86.3 (84.6–87.8)	46.8 (41.3–52.4)	61.1 (55.0–66.9)	90.2 (88.7–91.6)	49.7 (44.1 –55.2)	93.6 (92.3–94.7)	0.55 (0.49 –0.59)
ILD	92.1 (90.7–93.2)	47.8 (40.6–55.0)	51.3 (43.5–59.0)	95.8 (94.7–96.7)	53.0 (45.0 –60.8)	95.5 (94.4 –96.4)	0.52 (0.45 –0.59)

**Table 9:** Performance metrics for spirometry pattern, pattern severity and disease type classification.

Parameters	Overall Accuracy (%)	Macro-ave Precision (PPV)	Macro-ave Recall	Macro-aveSpecificity	Macro-aveF1-score	Cohen's Kappa
Spirometry Pattern	73.6 (71.9–75.3)	72.4	72.8	86.4	0.72	57.2
Pattern Severity	61.4 (59.6–63.3)	61.3	64.5	87.5	0.61	48.1
Disease Type	71.8 (69.7–73.8)	59.4	63.1	92.9	0.61	59.9

**Table 10:** Sensitivity and specificity at different Swaasa® thresholds for asthma detection.

Threshold	Sensitivity	Specificity
0.9	0.08	0.995
0.8	0.22	0.970
0.7	0.40	0.940
0.6	0.55	0.910
0.5	0.68	0.88
0.4	0.75	0.820
0.3	0.83	0.740
0.2	0.90	0.640
0.1	0.95	0.540

**Table 11:** Sensitivity and specificity at different Swaasa® thresholds for COPD detection.

Threshold	Sensitivity	Specificity
0.9	0.12	0.997
0.8	0.28	0.975
0.7	0.42	0.950
0.6	0.53	0.930
0.5	0.61	0.90
0.4	0.72	0.840
0.3	0.82	0.750
0.2	0.90	0.640
0.1	0.96	0.530

**Table 12:** Confusion matrix statistics for disease identification and spirometry abnormality with binary classification (differentiating normal and abnormal) with 95% confidence intervals.

Parameter	Sensitivity (%)	Specificity (%)	PPV (%)	NPV (%)	Accuracy (%)	Kappa (%)	Balanced Accura
Risk	92.3 (91.1–93.3)	80.7 (77.8–83.2)	92.6 (91.5–93.7)	79.9 (77.0–82.5)	89.10 (87.9–90.2)	72.70 (70.0–75.5)	0.865 (0.85–0.87)
Spirometry Pattern	85.3 (83.2–87.2)	73.8 (71.5–76.0)	72.7 (70.3–75.0)	86.0 (84.0–87.8)	79.00 (77.4–80.5)	58.20 (55.2–61.3)	0.796 (0.781–0.81)
Pattern Severity	82.2 (80.4–83.8)	60.9 (57.5–64.3)	83.7 (81.9–85.3)	58.4 (55.0–61.7)	76.0 (74.4–77.6)	42.6 (38.9–46.3)	0.716 (0.697–0.73)
Disease Type	93.4 (92.2–94.5)	82.6 (79.9–85.0)	92.1 (90.8–93.2)	85.3 (82.8–87.6)	90.00 (88.8–91.1)	76.70 (74.1–79.3)	0.880 (0.86–0.89)]

**Table 13:** Confusion matrix statistics for risk and pattern with binary classification at gender level with 95% confidence intervals.

Parameters	Accuracy (%)		Kappa Statistics (%)	
	Male	Female	Male	Female
Risk	88.2 (86.64–89.57)	90.6 (88.73–92.12)	68.6 (64.7–72.5)	78.2 (74.3–82.1)
Spirometry pattern	80.5 (78.51–82.30)	76.5 (73.77–78.97)	61.2 (57.4–65.0)	52.6 (47.3–57.8)

**Table 14:** Sensitivity and specificity for risk and pattern with binary classification at gender level with 95% confidence intervals.

Parameters	Sensitivity (%)		Specificity (%)		PPV (%)		NPV (%)		Balanced Accuracy	
	Male	Female	Male	Female	Male	Female	Male	Female	Male	Female
Risk	91.65 (90.08–92.99)	93.44 (91.48–94.98)	77.66 (73.64–81.22)	84.43 (80.36–87.78)	92.57 (91.07–93.84)	92.73 (90.70–94.35)	75.37 (71.3–79.03)	85.83 (81.85–89.06)	0.85 (0.82 −0.87)	0.89 (0.87 −0.91)
Spirometry Pattern	88.18 (85.75–90.24)	79.71 (75.60–83.29)	73.56 (70.56–76.36)	74.21 (70.57–77.55)	74.97 (72.10–77.64)	68.30 (64.05–72.27)	87.38 (84.8–89.58)	83.99 (80.63–86.87)	0.81 (0.79 −0.83)	0.77 (0.74 −0.79)

**Table 15:** Confusion matrix statistics for risk and pattern with binary classification at age level with 95% confidence intervals. .

Parameters	Accuracy (%)			Kappa Statistic (%)		
	18–47 yrs	48–63 yrs	64–89 yrs	18–47 yrs	48–63 yrs	64–89 yrs
Risk	85.6 (83.79–87.19)	92.2 (90.28–93.74)	95.6 (93.29–97.2)	69.5 (65.9–73.1)	73.2 (67.3–79.1)	73.0 (61.1–84.9)
Spirometry Pattern	77.2 (74.98–79.20)	79.8 (76.93–82.44)	84.5 (80.46–87.85)	52.3 (47.9–56.7)	59.2 (53.6–64.8)	61.2 (52.0–70.5)

**Table 16:** Confusion matrix statistics—accuracy and kappa statistics for risk, pattern, severity and disease variables with binary classification in recording 1, 2 and 3 with 95% confidence intervals.

Parameters	Accuracy (%)			Kappa Statistic (%)		
Recording No.	1	2	3	1	2	3
Risk	88.2 (86.5–89.8)	88.8 (86.7–90.7)	91.8 (88.9–94.0)	74.0 (70.4–77.7)	70.7 (65.5–75.8)	67.2 (57.2–77.1)
Spirometry Pattern	81.3 (79.1–83.3)	79.6 (76.8–82.1)	73.3 (68.8–77.4)	62.3 (58.1–66.5)	59.3 (54.0–64.6)	48.1 (40.2–56.0)
Pattern Severity	76.3 (73.9–78.5)	76.0 (73.1–78.7)	74.60 (70.1–78.6)	48.7 (43.8–53.6)	39.9 (32.5–46.1)	18.0 (6.9–29.0)
Disease Type	89.8 (88.0–91.3)	88.8 (86.5–90.7)	92.4 (89.3–94.6)	78.5 (75.1–81.9)	72.4 (67.3–77.5)	71.8 (62.1–81.2)

**Table 17:** Confusion matrix statistics—sensitivity and specificity for risk, pattern, severity and disease variables with binary classification in recording 1, 2 and 3 with 95% confidence intervals.

Parameters	Sensitivity (%)			Specificity (%)		
Recording No.	1	2	3	1	2	3
Risk	91.6 (89.6–93.2)	92.5 (90.4–94.2)	93.5 (90.6–95.6)	82.1 (78.5–85.1)	78.1 (72.6–82.8)	80.7 (68.7–88.9)
Spirometry Pattern	83.5 (80.2–86.3)	85.0 (81.2–88.1)	90.0 (84.7–93.6)	79.6 (76.6–82.3)	74.8 (70.7–78.6)	60.0 (53.5–66.2)
Pattern Severity	79.6 (76.8–82.2)	82.6 (79.5–85.4)	86.0 (81.8–89.4)	70.0 (65.7–74.0)	57.5 (51.0–63.7)	31.0 (22.1–41.5)
Disease Type	92.9 (90.9–94.5)	92.9 (90.6–94.7)	94.6 (91.6–96.6)	85.0 (81.7–87.8)	78.7 (73.2–83.3)	80.0 (68.2–88.2)

Please note the 845 mulit-lable observations with more than one clinical disease label were not included in the disease-specific classification analysis. These observations were included only in the normal-vs.-abnormal respiratory classification, for which the presence of one or more clinically established respiratory conditions was considered “abnormal.”

To evaluate the diagnostic accuracy of Swaasa® as a screening tool, confusion matrix statistics were employed. These statistics included accuracy, Kappa statistics, sensitivity, and specificity. Accuracy, denoting the proportion of correct predictions out of all predictions made, serves as a fundamental measure of overall model performance. The Kappa statistic assesses the agreement level between the predictive model and observed data. Sensitivity, or the true positive rate, quantifies Swaasa® 's ability to correctly identify true positive cases. Specificity, or the true negative rate, measures the model's capacity to accurately identify true negative cases (controls). These metrics collectively provide a comprehensive understanding of the model's proficiency in classifying outcomes. Diagnostic performance metrics (sensitivity, specificity, positive predictive value (PPV), negative predictive value (NPV), and accuracy) were calculated from the raw classification counts, and 95% confidence intervals (CIs) for these proportions were computed using the Wilson score method (29). For multiclass confusion matrices, 95% CIs for kappa were calculated using the large-sample standard error formula of Fleiss, Cohen, and Everitt (30). As F1-score has no closed-form standard error, its 95% CI was estimated using a percentile bootstrap: the class's true positive, false negative, false positive, and true negative counts were resampled 20,000 times from a multinomial distribution matching the observed proportions, and the 2.5th and 97.5th percentiles of the resulting F1 distribution were taken as the CI bounds. Because the model's continuous output scores were not available for normal vs. abnormal classification, a conventional ROC-AUC could not be computed. Instead, we report balanced accuracy, defined as (Sensitivity + Specificity)/2, at the model's single operating point. This is equivalent to the area under the two-segment ROC curve connecting (0,0), (1-Specificity, Sensitivity), and (1,1). 95% confidence intervals were obtained by propagating the variances of sensitivity and specificity using the delta method. Balanced accuracy is a single-operating-point metric and should not be interpreted as, or compared directly with, a conventional ROC-AUC. For the binary Asthma and COPD analyses, continuous model output scores were available. For these analyses, ROC curves were generated by varying the decision threshold, and the area under the curve (AUC) was calculated.

Confusion matrix statistics for risk, pattern, severity and disease with multi-class variables for complete data are in Table 8.

In addition, the Table 9 represents overall accuracy along with other parameters for the multiclass outputs.

Further, as an exploratory *post hoc* analysis, sensitivity and specificity were evaluated at different decision thresholds for asthma and COPD to characterize the sensitivity-specificity trade-off. For this analysis, each disease was treated as a binary one-vs.-rest classification problem, with the respective disease as the positive class and all other classes as the negative class. This threshold-based analysis was performed specifically for asthma and COPD, considering their clinical importance and prevalence in the study population.

The below Table 10 summarizes the threshold points and their associated sensitivity and specificity values for detecting the presence of asthma using Swaasa.

The Receiver Operating Characteristic (ROC) curve illustrating the diagnostic performance of Swaasa, along with the corresponding Area Under the Curve (AUC) value, is presented below. As Swaasa® is intended as a screening tool, a threshold of 0.3 emerged as a potentially suitable exploratory operating point providing high sensitivity while maintaining good specificity and an accuracy of 0.77, thereby reducing the likelihood of missing potential asthma cases. Figure 4 presents the receiver operating characteristic (ROC) curve for asthma.

**Figure 4:**
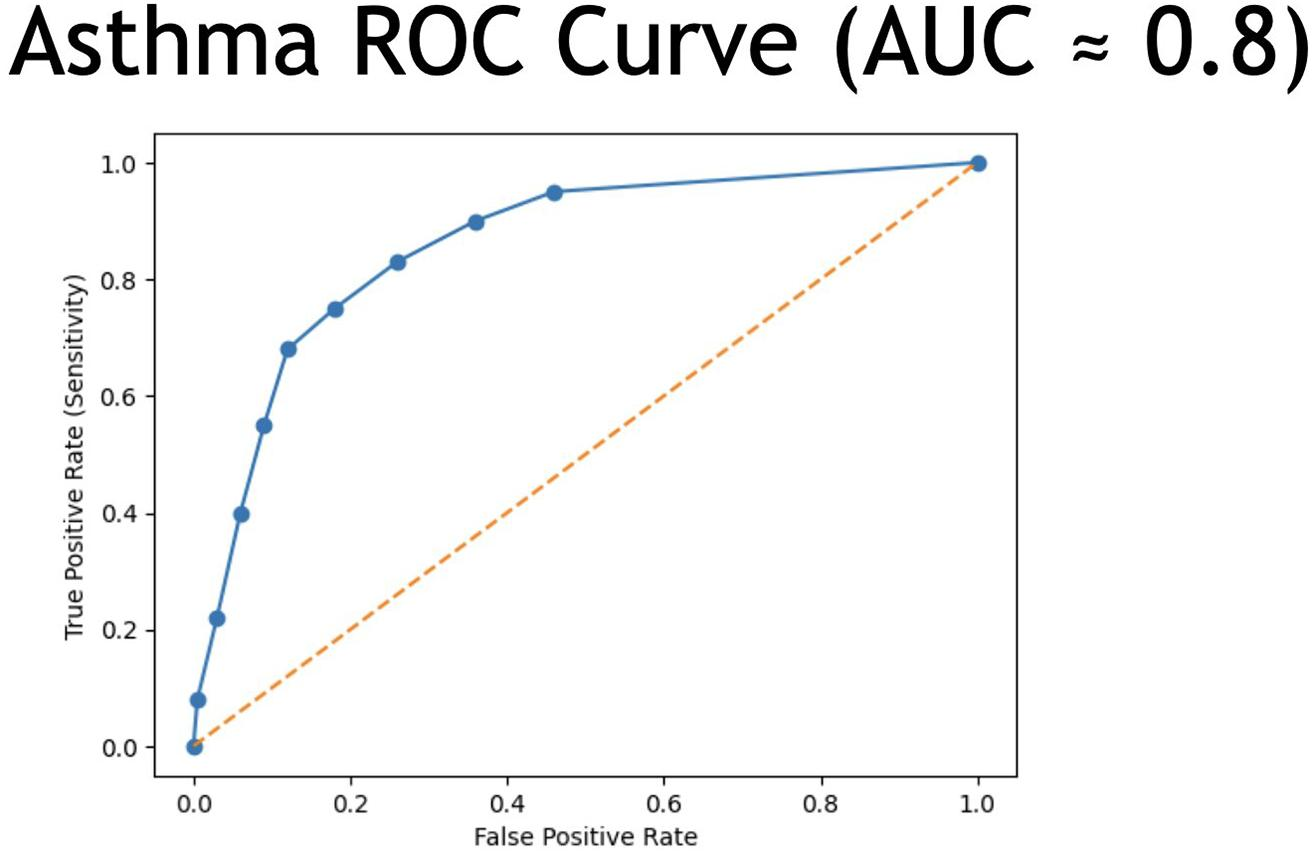
ROC curve for asthma.

The below Table 11 summarizes the threshold points and their associated sensitivity and specificity values for detecting the presence of COPD using Swaasa.

The Receiver Operating Characteristic (ROC) curve illustrating the diagnostic performance of Swaasa, along with the corresponding Area Under the Curve (AUC) value, is presented below. As Swaasa® is intended as a screening tool, a threshold of 0.3 emerged as a potentially suitable exploratory operating point providing high sensitivity while maintaining good specificity and an accuracy of 0.76, thereby reducing the likelihood of missing potential asthma cases. Figure 5 presents the receiver operating characteristic (ROC) curve for COPD.

**Figure 5:**
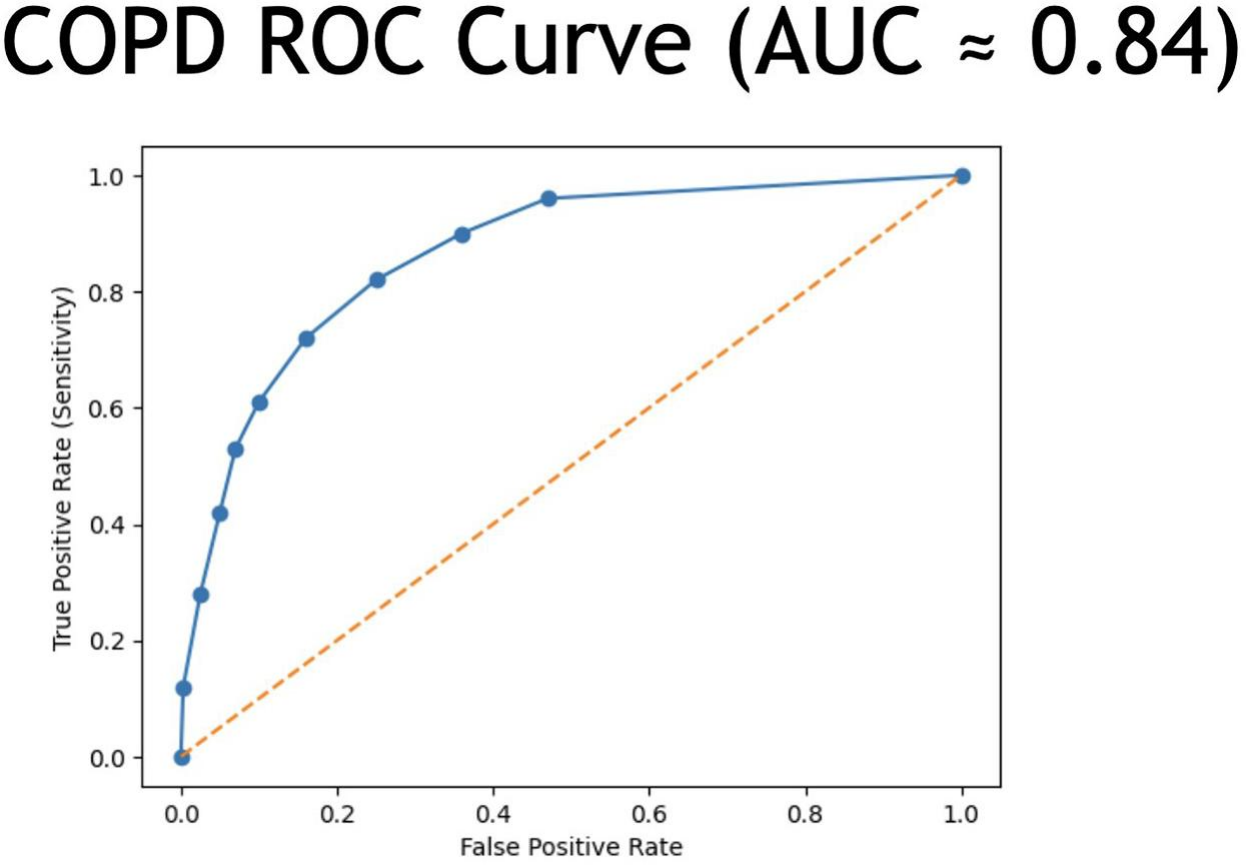
ROC curve for COPD.

As an exploratory *post hoc* analysis, multiple operating thresholds were evaluated to characterize the sensitivity-specificity trade-off for asthma and COPD. A threshold of 0.3 provided a potentially suitable balance for screening applications in the present dataset. This threshold was not prespecified before validation and should therefore be considered an exploratory operating point requiring prospective confirmation in an independent cohort.

Confusion matrix statistics for risk, pattern, severity and disease with binary classification for complete data are in Table 12, at the gender level are in Tables 13, 14, and at the sub-age level are in Table 15.

Exploratory subgroup performance by gender and age is presented in Tables 13–15. The purpose of these analyses was to explore whether there were substantial variations in model performance across demographic subgroups rather than to establish statistical equivalence between groups. Some numerical variation was observed across demographic subgroups. For spirometry-pattern classification, accuracy was 76.5% in females and 80.5% in males, while accuracy across age groups ranged from 77.2% to 84.5%. The observed performance was broadly comparable across gender and age subgroups. As no formal interaction testing was performed, these findings should not be interpreted as demonstrating statistical equivalence between subgroups.

The performance measures across Recordings 1, 2, and 3 are presented descriptively in Tables 16, 17. Sensitivity, specificity, and overall accuracy were broadly comparable across recordings for several assessed outcomes. These findings provide an assessment of algorithm performance across repeated cough acquisitions, while acknowledging variability across recordings for certain parameters, particularly pattern-severity agreement and specificity.

*This data was not used for validation.

No adverse events or unintended consequences related to the use of Swaasa® or the study procedures were observed during the study.

## Discussion

The Swaasa® AI platform is intended to complement established clinical assessment and diagnostic methods. This study demonstrates the feasibility of smartphone-based cough acquisition and analysis and evaluates its performance for respiratory screening and clinical assessment.

The non-invasive, smartphone-based approach requires limited additional infrastructure and may support wider use in primary care, community, remote, and resource-constrained settings. By identifying individuals at risk of respiratory abnormalities, Swaasa® may also help prioritize individuals for further clinical evaluation and confirmatory testing. However, these potential applications were not directly evaluated in this study. Further studies are required to assess real-world implementation, cost-effectiveness, workflow impact, and patient outcomes.

Previous studies have investigated cough-sound analysis for respiratory conditions including asthma, pneumonia, COPD, and bronchitis (10, 29–31). The COVID-19 pandemic further accelerated research into machine-learning approaches using cough sounds for respiratory screening (3,5), supporting continued investigation of cough acoustics as a non-invasive source of clinically relevant information.

The observed concordance between the Swaasa AI platform and spirometry highlights the potential of AI-driven tools to support respiratory screening and clinical assessment in primary healthcare. The observed performance for COPD and asthma suggests that cough sound analysis may help identify individuals at risk of obstructive respiratory conditions and support further clinical evaluation, particularly in settings where access to spirometry is limited.

The Swaasa AI platform offers several advantages for respiratory screening and clinical assessment. Its non-invasive, software-based approach enables assessment using widely available devices such as smartphones, reducing dependence on specialized equipment at the initial screening stage. Moreover, the platform's ease of use and minimal training requirements make it an attractive option for rural healthcare centers, where trained personnel and conventional respiratory assessment tools are often scarce.

## Limitations

Despite the rigorous methodology, several limitations must be acknowledged. Respiratory disease classification relied on clinician assessment using multidisciplinary discussions, guideline-based approaches (e.g., GOLD, GINA), and radiology, but overlapping features (e.g., asthma–COPD overlap, cough from GERD) and coexisting conditions (e.g., COPD with bronchiectasis) may have introduced confounding. Spirometric abnormality was mapped to FEV₁ categories for severity, which may be suboptimal for diseases such as asthma or ILD, where FVC or DLCO may better reflect severity. The device did not evaluate all potential causes of cough (e.g., post-nasal drip, GERD, respiratory infections, tumours), and dedicated studies are required for these. Finally, as this was a single-center study in a tertiary-care hospital, multi-center studies in primary care and community settings are needed before broader clinical implementation.

The validation population was recruited from a tertiary-care hospital and may not fully represent the general community population. The normal controls were asymptomatic relatives of patients, and the study included a higher proportion of patients with respiratory diseases than would be expected in routine community screening. Therefore, the reported performance, particularly predictive values, may not directly generalize to primary-care or community settings. Further studies in community settings are needed to confirm these findings.

While the pooled analyses include repeated recordings from individual participants and therefore do not fully account for within-participant correlation, the Record 1 analysis in Tables 16, 17 provides participant-level performance estimates based on one independent observation per participant.

## Conclusion

This paper presents a deployable AI-based tool for preliminary screening of and clinical assessment of respiratory diseases such as asthma, COPD, ILD, and bronchiectasis using cough sounds as a marker. The approach builds on our prior work demonstrating that cough sounds contain clinically relevant acoustic features that support identification of differentiation of respiratory conditions. Because pathological processes affect the respiratory system in distinct ways, the associated cough is expected to exhibit specific latent features. Leveraging these clinical insights, we developed the Swaasa AI platform for cough-based screening and clinical assessment tools, and our results indicate that it can identify respiratory conditions without requiring conventional clinical infrastructure.

## Data Availability

The datasets presented in this article are not readily available because Due to the nature of this research, participants of this study did not agree for their data to be shared publicly. Requests to access the datasets should be directed to svn@salcit.in, shubhadeepti@salcit.in.
