# Supplementary material for: Clinical validation of the Swaasa® artificial intelligence platform using cough sounds for screening and aid in diagnosis of respiratory diseases
Source: Front Med (Lausanne). 2026 Oct 28;13:1964851. doi: 10.3389/fmed.2026.1964851 (PMC13751465; doi:10.3389/fmed.2026.1964851)
Supplement: Supplementary file 1 [file Table1.docx]

**Supplementary Material**

The manuscript was assessed against the 30-item STARD 2015 checklist.

| **STARD item** | **Requirement** | **Status** | **Manuscript location / evidence** |
| --- | --- | --- | --- |
| 1 | Identification as a diagnostic accuracy study using ≥1 accuracy measure | **Yes** | Title, Abstract |
| 2 | Structured summary of design, methods, results and conclusions | **Partial** | Abstract |
| 3 | Scientific/clinical background, intended use and clinical role of index test | **Yes** | Introduction, pp. 2–3 |
| 4 | Study objectives and hypotheses | **Partial** | Introduction, p. 3; Study design, p. 5 |
| 5 | Whether data collection was planned before index/reference tests (prospective or retrospective) | **Yes** | Abstract; Study design, p. 5 |
| 6 | Eligibility criteria | **Partial** | Data collection/Methods, pp. 3–4 |
| 7 | Basis on which potentially eligible participants were identified | **Partial** | Methods, pp. 3–4 |
| 8 | Where and when potentially eligible participants were identified | **Partial** | Ethics/Study design, pp. 4–5; trial registration |
| 9 | Consecutive, random or convenience series | **Yes** | Methods, p. 4 |
| 10a | Index test in sufficient detail for replication | **Yes** | Introduction pp. 2–3; Methods pp. 5–6 |
| 10b | Reference standard in sufficient detail for replication | **Yes** | Methods, p. 4 |
| 11 | Rationale for choosing reference standard | **Yes** | Methods, p. 4 |
| 12a | Definition/rationale for index-test positivity cut-offs/result categories; prespecified vs exploratory | **Partial** | Methods pp. 5–6; Results pp. 14–16 |
| 12b | Definition/rationale for reference-standard positivity/result categories; prespecified vs exploratory | **Partial** | Methods pp. 4, 9 |
| 13a | Whether clinical information/reference-standard results were available to index-test performers/readers | **Partial** | Methods, p. 4; p. 5 |
| 13b | Whether clinical information/index-test results were available to reference-standard assessors | **Yes** | Methods, p. 4 |
| 14 | Methods for estimating/comparing diagnostic accuracy | **Yes** | Results statistical methods, p. 12; Tables 8–17 |
| 15 | Handling of indeterminate index/reference-standard results | **Partial** | Methods pp. 3–4; Results pp. 8–12 |
| 16 | Handling of missing index/reference-standard data | **Partial** | Results Tables 2–7; p. 12 |
| 17 | Analyses of variability in diagnostic accuracy, prespecified vs exploratory | **Partial** | Results pp. 16–18 |
| 18 | Intended sample size and how determined | **Yes** | Methods, pp. 4–5 |
| 19 | Flow of participants using a diagram | **Partial** | Results, p. 7 |
| 20 | Baseline demographic and clinical characteristics | **Yes** | Table 1, p. 7; Figure 3 |
| 21a | Distribution of disease severity among participants with target condition | **Partial** | Tables 3, 6, 8–9 |
| 21b | Distribution of alternative diagnoses among participants without target condition | **Partial** | Tables 2–3; Limitations |
| 22 | Time interval and clinical interventions between index test and reference standard | **Yes** | Methods, p. 3 |
| 23 | Cross-tabulation of index-test results by reference-standard results | **Yes** | Tables 4–7, pp. 10–12 |
| 24 | Diagnostic accuracy estimates and precision | **Yes** | Tables 8–17, pp. 13–18 |
| 25 | Adverse events from index/reference-standard procedures | **Yes** | Results, p. 19 |
| 26 | Study limitations including bias, statistical uncertainty and generalisability | **Yes** | Limitations, pp. 19–20 |
| 27 | Implications for practice, intended use and clinical role | **Yes** | Discussion/Conclusion, pp. 19–20 |
| 28 | Registration number and registry name | **Yes** | Abstract; Ethics, p. 4 |
| 29 | Where full study protocol can be accessed | **Yes** | Ethics, p. 4 |
| 30 | Funding/other support and role of funders | **Yes** | Funding and Conflicts of Interest, p. 21 |
